# Selective Janus Kinase 1 Inhibition Is a Promising Therapeutic Approach for Lupus Erythematosus Skin Lesions

**DOI:** 10.3389/fimmu.2020.00344

**Published:** 2020-03-03

**Authors:** Tanja Fetter, Paul Smith, Tugce Guel, Christine Braegelmann, Thomas Bieber, Joerg Wenzel

**Affiliations:** ^1^Department of Dermatology and Allergy, University Hospital Bonn, Bonn, Germany; ^2^Incyte Corporation, Wilmington, DE, United States

**Keywords:** lupus erythematosus, skin, kinase inhibitor, innate immunity, interferon

## Abstract

**Background:**

Cutaneous lupus erythematosus (CLE) is an interferon (IFN) -driven autoimmune skin disease characterized by an extensive cytotoxic lesional inflammation with activation of different innate immune pathways. Aim of our study was to investigate the specific role of Janus kinase 1 (JAK1) activation in this disease and the potential benefit of selective JAK1 inhibitors as targeted therapy in a preclinical CLE model.

**Methods:**

Lesional skin of patients with different CLE subtypes and healthy controls (*N* = 31) were investigated on JAK1 activation and expression of IFN-associated mediators via immunohistochemistry and gene expression analyses. The functional role of JAK1 and efficacy of inhibition was evaluated *in vitro* using cultured keratinocytes stimulated with endogenous nucleic acids. Results were confirmed *in vivo* using an established lupus-prone mouse model.

**Results:**

Proinflammatory immune pathways, including JAK/STAT signaling, are significantly upregulated within inflamed CLE skin. Here, lesional keratinocytes and dermal immune cells strongly express activated phospho-JAK1. Selective pharmacological JAK1 inhibition significantly reduces the expression of typical proinflammatory mediators such as CXCL chemokines, BLyS, TRAIL, and AIM2 in CLE *in vitro* models and also improves skin lesions in lupus-prone TREX1^–/–^ -mice markedly.

**Conclusion:**

IFN-associated JAK/STAT activation plays a crucial role in the pathophysiology of CLE. Selective inhibition of JAK1 leads to a decrease of cytokine expression, reduced immune activation, and decline of keratinocyte cell death. Topical treatment with a JAK1-specific inhibitor significantly improves CLE-like skin lesions in a lupus-prone TREX1^–/–^ -mouse model and appears to be a promising therapeutic approach for CLE patients.

## Introduction

Cutaneous lupus erythematosus (CLE) is an inflammatory autoimmune skin disease with heterogenous subtypes varying from localized discoid plaques to severe and widespread erythro-squamous lesions in affected patients (1). The disease is characterized by a typical immunohistological pattern called “interface dermatitis” (ID) consisting of colloid bodies and an anti-epidermal cytotoxic lymphocytic infiltrate in the dermo-epidermal junction which is orchestrated by IFN-regulated proinflammatory cytokines (2).

Cutaneous lupus erythematosus is of multifactorial origin, including both genetic and environmental risk factors (3, 4). In particular UV radiation promotes cellular damage, apoptosis and release of DNA-containing blebs. It is considered that cell debris clearance is impaired entailing secondary necrosis (5, 6). In some cases such as familial chilblain lupus the underlying cause can be TREX1 gene mutations leading to a dysfunctional TREX1 exonuclease and high accumulation of cytosolic DNA (7, 8).

**FIGURE 1:**
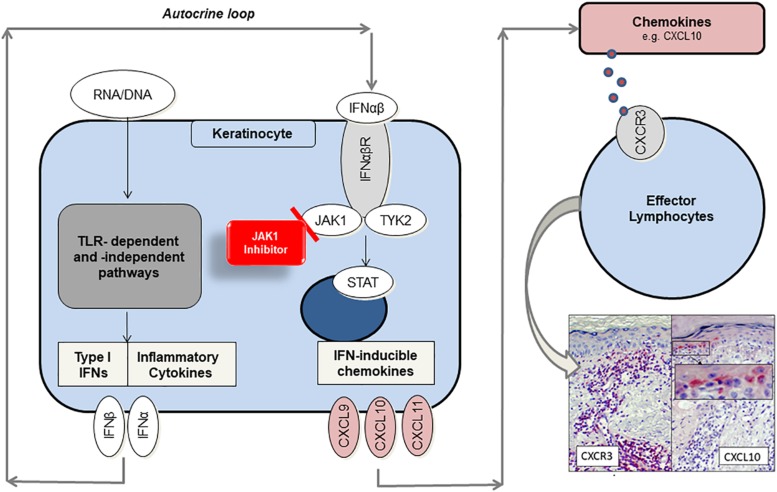
Proposed role of JAK1 and function as target of small-molecule inhibitors in CLE. Endogenous nucleic acids (RNA/DNA) activate TLR-dependent and –independent pathways to produce proinflammatory cytokines and type I interferons (IFN). In an autocrine loop IFNs activate JAK1/TYK2-signaling by binding to IFN-α/β receptor, thus enhancing CXCL chemokine production (e.g., CXCL10). Chemokines recruit CXCR3 + effector cells into lesional skin, which induce keratinocyte cell death and again release of proinflammatory mediators. JAK1-inhibitors prevent JAK/STAT-mediated signal transduction and significantly reduce chemokine gene expression by functionally targeting JAK1.

When nuclear components, such as endogenous nucleic acid motifs, are released out of the nucleus due to cellular damage, they can be perceived as danger associated molecular patterns (DAMPs) (9). There is evidence that keratinocytes and particularly plasmacytoid dendritic cells (pDCs) react on DAMPs inappropriately with immense type I IFN production through Toll-like receptor (TLR)-dependent or – independent pathways in CLE (10–12). Undergoing an autocrine loop IFNs bind to IFN-α/β receptors on keratinocytes, thus activating JAK/STAT (Janus kinase/signal transducer and activator of transcription) pathway and expression of proinflammatory mediators such as CXCL10. It is known that CXCL10 recruits CXCR3 + effector cells and pDCs into skin lesions (2). Those effector cells induce keratinocyte necroptosis, cytokine release and a continuous “self-recruitment” as a hallmark of chronic inflammation (Figure 1) (13).

This vicious circle can result in a strong burden for quality of life (14, 15). Following current guidelines corticosteroids and antimalarials are established as first-line treatment of CLE. However, long term therapy with glucocorticosteroids is limited by side effects and, particularly in antimalarial-resistant patients, can be challenging. In addition still no specifically approved drugs exist and only a few clinical trials have been conducted, not least because of clinical heterogeneity and thus challenging trial design (16). Considering these limitations there is a strong unmet medical need for new therapeutic options.

Recent insights into the molecular pathogenesis of CLE led to the development of therapeutic strategies targeting molecules or immune cells of innate and adaptive immune responses. As the type I IFN signature plays a key role in LE, novel compounds were developed affecting the IFN-system and JAK/STAT signaling. Currently oral JAK inhibitors are investigated in systemic lupus erythematosus (SLE) patients in clinical trials (17). First clinical reports suggested a benefit of oral JAK1/2 inhibitors on CLE skin lesions, but the systemic use of these drugs is limited by side effects, e.g., anemia and thrombopenia, which mainly depend on JAK2 blockade. This prompted us to perform this preclinical study to evaluate the use of topical JAK1 selective inhibition as a new therapeutic principle in CLE.

## Patients and Methods

### Patients and Skin Samples

All punch biopsies of the different inflammatory skin disorders (*N* = 22) were taken for diagnostic purposes from active skin lesions. Healthy controls (*N* = 9) were taken from unaffected skin taken from plastic surgery. Skin samples were fixed with 4% formalin over night or fixed in frozen nitrogen and proceeded for immunohistochemistry or RNA isolation. RNA was processed by the next generation sequencing (NGS) Core Facility of the Medical Faculty of the University of Bonn using the QuantSeq 3′-mRNA Library Prep Kit by Lexogen. Illumina HiSeq 2500 was used for RNA sequencing (Standard 3′RNA seq with 50 cycles). This study was performed in accordance to the principles of the Declaration of Helsinki and approved by the local Ethics Committee in Bonn (BN 09004).

### Immunohistochemistry

Samples of lesional skin from CLE patients were H&E stained to confirm the clinical diagnosis in every single case by an experienced dermatopathologist (JW). Immunohistochemistry was performed using the REAL Detection Systems with Fast Red as chromogen (Agilent, Santa Clara, CA, United States) with specific antibodies for pJAK (ABIN196869, antibodies-online), CXCL10 (ab9807, Cambridge, United Kingdom), and CD45 (550539, BD, New Jersey). The expression was scored semiquantitatively from 0 =̂ weak to 3 =̂ strong (18). Immunofluorescence analyses of JAK1-phosphorylation detected by anti-rabbit Rhodamine Red-X (711-295-152; Jackson ImmunoResearch, Baltimore, MD, United States) and DAPI (D9542, Sigma-Aldrich) were performed using a high-resolution microscope (Axio Observer Z1, Zeiss, Germany).

### Cell Culture Experiments

Immortalized keratinocytes (HaCaT), were acquired from CLS Cell Lines Service GmbH, Eppelheim, Germany), normal human epidermal keratinocytes (NHEKs, FC-0025) and Human epidermis equivalents (epiCS, CS-1001) from CellSystems, Troisdorf, Germany. These cell lines were cultured according the manufactures protocols. Cultured keratinocytes were stimulated with endogenous nucleic acids (eNA, 12,5 μg/mL) isolated from unstimulated keratinocytes using the “Genomic DNA from tissue” kit (Machery-Nagel, Dueren, Germany). Lipofectamine 2000 (Invitrogen, Carlsbad, CA, United States) functioned as a transfection reagent (2,5 μl/mL). INCB039110 provided by Incyte, Wilmington, DE, United States, as well as Ruxolitinib (Selleckchem, Eching, Germany) were added at a final concentration of 1 μM; JAK3 selective FM-381 was used as recommended (100 nm) (19). All experiments were implemented in biological triplicates. Enzyme-linked immunosorbent assays for human CXCL10 (DY266-05 R&D systems) were performed using DuoSet Ancillary Reagent Kit 2 (DY008 R&D systems) according to the supplied protocol, measured by Synergy HT Multi-Detection Multiplate Reader (BioTek, Winooski, VT, United States) and read out with Gen5 software (version 1.11.5).

### Murine Experiment

TREX1^–/–^ mice (generated on C57BL/6J background by Thomas Lindahl, Cancer Research Institute, London, United Kingdom) were bred and maintained under specific pathogen-free conditions at the animal core facility of UKB Bonn (HET, Bonn, Germany). The animal experiment was performed in accordance with the guidelines of the EU Directive 2010/63/EU and approved by the Animal Welfare Commission of North Rhine-Westphalia, Germany (AZ 2014.A436). TREX1^–/–^ mice (*n* = 8) were back-shaved and treated with 0,2% DNFB (1-Fluor-2,4-dinitrobenzol, Sigma Aldrich). 4 days later UV-irradiation on 3 sequential days started with 450 mJ/cm^2^ UVB for 115 s per day using UV801KL (Waldmann, Villingen-Schwenningen, Germany). For 7 days 1% INCB039110 or placebo solved in DMSO and olive oil (50 μl per mouse) were applied topically. Every day photos of mice were taken and every 2 days mice were weighed.

### Statistical and Gene Expression Analyses

All statistical analysis of *in vitro* experiments were performed with GraphPad prism software (version 7) using Kruskal-Wallis-Test and Mann-Whitney *U* test. Gene expression was analyzed with Partek Flow genomic analysis software and Subio Platform software v1.22.5266 using Welch’s *t*-test. Confidence intervals were determined at 95%. *P* < 0.05 was considered to be “significant” (^∗^), *p* < 0.01 to be “highly significant” (^∗∗^). KEGG pathways were mapped to differentially expressed genes using DAVID v6.8 (Database for Annotation, Visualization and Integrated Discovery).

## Results

### Activated JAK1 Is Strongly Expressed in Human CLE Skin Lesions

**FIGURE 2:**
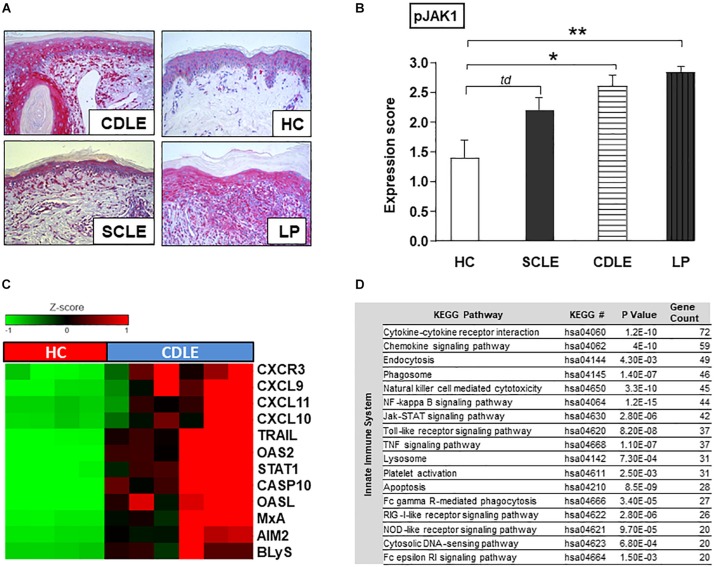
Functional role of JAK1 expression in CLE skin lesions. **(A)** Representative immunohistochemical sections (anti-phospho JAK1 staining) from skin samples of patients with different inflammatory skin disorders [Subacute cutaneous lupus erythematosus (SCLE, *n* = 5), chronic discoid lupus erythematosus (CDLE, *n* = 5), Lichen planus (LP, *n* = 6), and healthy controls (HC, *n* = 5)], original magnification ×200. **(B)** The expression of pJAK1 was scored semi-quantitatively by scoring from 0 =̂ no expression to 3 =̂ strong expression (18). All bars show mean ± SEM, td =̂ *p* < 0.1, * =̂ *p* < 0.05, ** =̂ *p* < 0.01 (Kruskal-Wallis-Test). **(C)** Upregulated expression of IFN-associated genes in CLE lesional skin (twofold, *p* < 0.01; Welch’s *t*-test) compared to healthy controls (HC). **(D)** KEGG pathways upregulated in CLE. KEGG pathway classification was performed using Database for Annotation, Visualization and Integrated Discovery (DAVID version 6.8). *P*-values were generated with EASE Score. Count: number of genes >twofold upregulated in CLE within the respective KEGG pathway.

To investigate the specific role of JAK1-mediated signaling in CLE, phosphorylated JAK1 (pJAK1) expression in lesional skin [Subacute cutaneous lupus erythematosus (SCLE, *n* = 5) and chronic discoid lupus erythematosus (CDLE, *n* = 5) subsets] was compared to lichen planus (LP, *n* = 6), another inflammatory skin disorder, as well as healthy controls (HC, *n* = 5). In CLE skin lesions the expression of pJAK1 was significantly increased in keratinocytes from stratum basale to stratum granulosum and in dermal infiltrating immune cells. Interestingly, pJAK1 was also significantly enhanced in LP, an autoimmune disease sharing common histological features with CLE (Figures 2A,B).

### JAK/STAT-Associated Innate Inflammatory Pathways Are Significantly Activated in Human CLE Skin

Within CLE lesional skin (*n* = 6) gene expression analysis revealed a significant activation of genes associated with both innate and adaptive immune pathways compared to healthy controls (*n* = 4) (Supplementary Figure S1). In particular genes of LE-associated proinflammatory chemokines (CXCL10,9,11) and other IFN-regulated proteins (OASL, OAS2, and MxA) as well as key drivers in cell death and B-cell activation (CXCR3, CASP10, AIM2, TRAIL, and BLyS) were highly expressed. As transcription of many genes is mediated by JAK/STAT signaling, gene expression of STAT1 was also significantly increased (Figure 2C). Upregulated innate immune pathways, in which aforementioned associated highly expressed genes play crucial roles, included JAK/STAT pathway and cytokine-/chemokine signaling as well as upstream TLR-dependent and -independent DAMP-recognition pathways (Figure 2D).

### INCB039110 Significantly Inhibits JAK1-Phosphorylation in Cultured Immortalized Human Keratinocytes

To analyze the functional principle and effect of JAK inhibition, established *in vitro* models of CLE were used. Cultured keratinocytes were stimulated with endogenous nucleic acids (eNA) and treated with inhibitors afterward.

**FIGURE 3:**
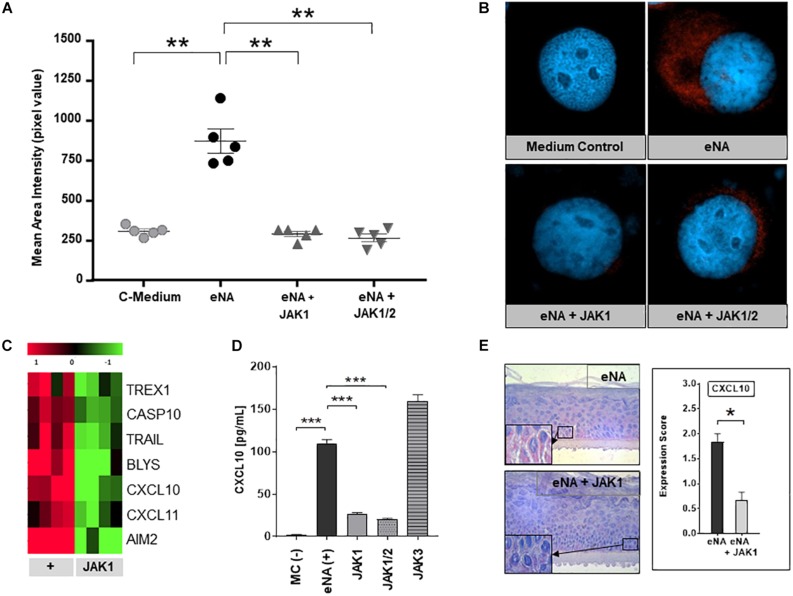
Effects of pharmacological JAK1 inhibition on proinflammatory cytokine expression and IFN-associated gene regulation in cultured keratinocytes. **(A)** The graph shows mean area intensity in pixel values of anti-pJAK1-antibody staining in stimulated HaCaT (eNA), stimulated and inhibitor-treated HaCaT (eNA + JAK1, inhibitor:INCB039110); eNA + JAK1/2, inhibitor:ruxolitinib and unstimulated HaCaT (C-Medium). Measurement was performed out using Fiji software. Measurements per entity: n = 5. All scatter dot plots show mean + SEM, ** =̂ *p* < 0.01 (Mann-Whitney *U* test). **(B)** Representative immunofluorescence micrographs of eNA-stimulated untreated (eNA) or inhibitor-treated (eNA + JAK1, inhibitor:INCB039110; eNA + JAK1/2, inhibitor:ruxolitinib) HaCaT as well as unstimulated HaCaT-cells (Medium Control). Anti-pJAK1 antibody staining in red, DAPI staining of the nucleus in blue, original magnification ×400. **(C)** Expression of CLE-typical genes within eNA-stimulated NHEK (+, 4 samples) and >twofold downregulated expression (*p* < 0.05; Welch’s *t*-test) after treatment of stimulated NHEK with JAK1 selective INCB039110 (JAK1; 4 samples). **(D)** Effect of different JAK inhibitors on CXCL10 expression in eNA-stimulated HaCaT after 24 h of incubation. Cells were treated with JAK1 selective INCB039110 (JAK1), JAK1/2 selective Ruxolitinib (JAK1/2), and JAK3 selective FM-381 (JAK3). Unstimulated cells [MC(-)] functioned as medium control and eNA-stimulated, but not inhibitor-treated cells [eNA(+)] as positive control. Measured by ELISA. All bars show mean + SEM, *** =̂ *p* < 0.001, ** =̂ *p* < 0.01 (Mann-Whitney *U* test). **(E)** Micrographs representing the expression of CXCL10 in stimulated human “epiCS” epidermis equivalents (eNA, *n* = 3) and JAK1 inhibitor treated cells (INCB039110) after stimulation (eNA + JAK1, *n* = 3), original magnification ×400, with corresponding CXCL10 expression score. All bars show mean ± SEM, * =̂ *p* < 0.05 (Mann-Whitney *U* test).

JAK1-phosphorylation was strongly enhanced within HaCaT after stimulation with eNA, corresponding with our findings in CLE skin lesions. As depicted in Figures 3A,B, JAK1-specific INCB039110 and JAK1/2-specific ruxolitinib significantly decreased the activation of JAK1 within stimulated cells.

### Pharmacological JAK1 Inhibition Blocks the Expression of CLE-Typical Proinflammatotry Cytokines and Pathway Molecules *in vitro*

To investigate the efficacy of pharmacological JAK1 inhibition, *in vitro* analyses in three different CLE-models were performed (i) HaCaT-cells, (ii) NHEK-cells, and (iii) 3D epidermis equivalents. As demonstrated in Figure 3C, pharmacological JAK1 inhibition induced a significant downregulation of genes encoding key drivers of innate inflammatory pathways such as IFN-regulated chemokines (CXCL10, CXCL11), cell death (TRAIL, AIM2, and TREX1) and cross-talk to adaptive immune cells (BlyS) within primary keratinocytes (NHEK) compared to untreated eNA-inflamed cells. Associated downregulated KEGG pathways are listed in Supplementary Table S1. These results were consistent with findings in HaCaT-cells, where both JAK1- and JAK1/2 inhibitors decreased the protein expression of CXCL10 significantly. Interestingly, inhibition of JAK3 did not obtain a reduction of CXCL10 expression (Figure 3D and Supplementary Figures S2A,B). In 3D epidermis equivalents, exposure of a JAK1 selective inhibitor to stimulated epiCS revealed a significantly reduced CXCL10 protein expression, conforming with findings described above (Figure 3E and Supplementary Figure S2C).

### *In vivo* Topical Application of INCB039110 Ameliorates CLE-Like Lesions in Lupus-Prone TREX1^–/–^ Mice

**FIGURE 4:**
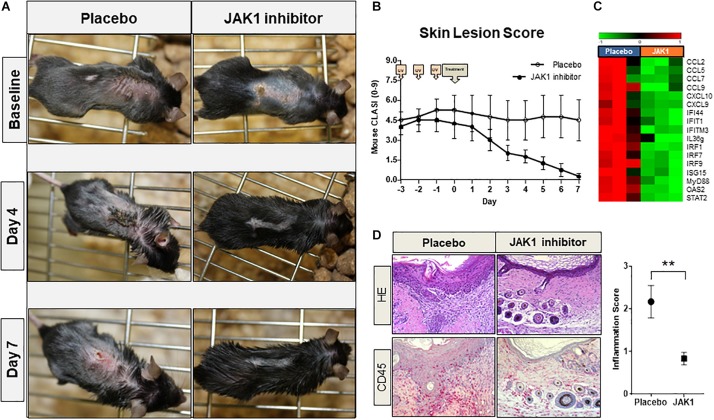
Pharmacological JAK1 inhibition in vivo in a lupus-prone TREX1^–/–^ mouse model (using JAK1 inhibitor INCB039110). **(A)** Clinical findings of UV-stimulated (started on day -3; 3 × 450 mJ/cm^2^ for 115 s) TREX1^–/–^ mice (n = 8) on baseline (day 0, start of treatment) and after 4 and 7 days of treatment with JAK1 selective INCB039110 or placebo. **(B)** Mean score of the mouse-adapted revised CLE-disease-area-and-severity index (Mouse RCLASI) ± SEM in n = 4 mice per group. **(C)** IFN-associated differentially expressed genes in lesional skin of TREX1^–/–^ mice treated with a JAK1 inhibitor (JAK1) or placebo, showing a significant downregulation after JAK1 inhibitor treatment (>twofold, *p* < 0.05,Welch’s *t*-test). **(D)** Histological (H&E) and immunohistological (CD45) micrographs of lesional skin, original magnification ×400. Corresponding inflammation score of CD45 expression in lesional skin of placebo-treated and JAK1-inhibitor treated mice. All bars show mean ± SEM, ** =̂ *p* < 0.01 (Mann-Whitney *U* test).

TREX1^–/–^ mice spontaneously developed CLE-like erythrosquamous and partly ulcerated skin lesions at a certain age which intensified after UVB-provocation. Topical treatment with JAK1-specific INCB039110 for 7 days continuously improved skin lesions regarding erythema, induration, scaling and size leading to a strongly reduced lupus-skin-activity-score (adapted CLASI score) compared to placebo-treated mice (Figures 4A,B). In addition, IFN-associated genes including CXCL and CCL chemokines, IFN regulatory factors (IRF) and interferon induced (IFIT) family genes were significantly downregulated in JAK1 inhibitor treated lesional skin (Figure 4C), consistent with notably improved distinct histological features such as epidermal thickness and infiltrating dermal immune cells (Figure 4D).

## Discussion

First evidence for a functional role of type I IFNs in LE came from clinical observations in patients suffering from carcinoid tumors which were treated with recombinant IFNα and developed SLE due to this therapy (20). This idea was strongly supported by findings in multiple sclerosis patients who developed CLE-like lesions at the injection side of recombinant IFNβ after subcutaneous application (21). Most importantly gene expression analyses in both SLE and CLE revealed a strong expression of type I IFN associated proinflammatory cytokines in blood and skin in association with disease activity (22). These earlier findings are also supported by our results, demonstrating a strong expression of IFN-regulated genes and immune pathways within CLE skin lesions (Figure 2).

Earlier data revealing strong IFN-signatures prompted the development of anti-IFN directed therapeutic strategies of LE patients. Here, anti-IFNα-agents (e.g., sifalimumab and rontalizumab) were the first drugs tested in clinical studies. These agents reduced the IFN signature in the blood but had limited effect on disease activity. This was possibly due to the high variability of the different type I IFNs, including not only IFNα but also IFNβ and IFNκ binding to the same receptors (23–25). Accordingly, targeting the common receptor was more effective: the anti-IFNαβ-receptor antibody anifrolumab significantly reduced the CLASI skin score in SLE patients in a recent clinical study (26). Alternative strategies focused on “indirect” inhibition of the IFN system by (i) targeting the IFN-producing cells or (ii) the intracellular IFN-pathway. In skin and blood plasmacytoid DCs are regarded as the main IFN-producing cells (27) and the pDC-specific antibodies BIIB059 and VIB7734 are now in clinical trials with first results indicating a decline of the CLASI-activity score (2, 28). With regards to the IFN-pathway, particularly JAK inhibitors were in the focus of recent emerging concepts.

The JAK family consists of four non-receptor tyrosine kinases (JAK1-3, TYK2) that transduce signals from growth factors and cytokines such as type I/III IFNs. JAK inhibitors were initially developed for the treatment of hematologic diseases with definite JAK-mutations showing anticlonal activity (29, 30), but they also provide significant immunosuppressive effects (31–34).

Earlier studies revealed a strong activation of JAK/STAT pathway and JAK protein expression in CLE and associated skin disorders such as lichen planus (LP), which is supported by our data (Figure 2) (9). Remarkably, LP is characterized by a quite similar pathogenesis: Strongly upregulated IFNs initiate JAK/STAT mediated CXCL chemokine expression (particularly CXCL9,10,11) in keratinocytes resulting in a typical ID pattern with epitheliotropic cytotoxic effector cells and cell death of basal keratinocytes (35). Accordingly, JAK inhibitors provided beneficial effects in diseases featuring ID including graft-versus-host disease (36) and dermatomyositis (37) besides other inflammatory skin conditions (38). In CLE, JAK1/2 inhibitors ruxolitinib (39, 40) and baricitinib (41) as well as JAK1/3 inhibitor tofacitinib (42) have been reported to be successful in patient’s treatment, and these drugs significantly decrease the expression of CLE-typical chemokines *in vitro* (43, 44).

One crucial disadvantage of these drugs are their side effects, including anemia and thrombopenia, mainly depending on JAK2- and JAK3 inhibition, which prompted us to focus on JAK1 in this preclinical project (32). Our *in vitro* data demonstrates that selective JAK1 inhibitors are as potent as JAK1/2 inhibitors in suppression of CLE typical cytokines (Figure 3D) and prohibit gene expression encoding proinflammatory chemokines (CXCL10-11), lymphocyte activators (BLyS) and cell death promotors (TRAIL, AIM2, Caspase 10, and TREX1). Interestingly, inhibition of JAK3 was not effective on CXCL10, which is a central mediator of CLE-typical ID. This could explain the earlier failure of the JAK3/SYK blocking agent R333 in a clinical CLE study (45). A central role for JAK1 in CLE-associated immune signaling also is supported by recent findings of mice developing CLE-like skin lesions due to JAK1-hyperactivation (46). Currently, a phase II clinical trial investigates the efficacy of orally administered JAK1 inhibitor filgotinib in female patients with active CLE (NCT03134222). In SLE patients, JAK1 inhibitor GSK2586184 had no significant effect on IFN transcriptional biomarker expression compared to placebo in a phase II clinical study, most probably due to limitations by low numbers of patients (47). A clinical study with JAK1/2 inhibitor baricitinib, however, revealed significantly improved SLE symptoms, supporting a critical role of JAK/STAT signaling in the pathogenesis of SLE (48). As activated JAK1 elicits signals from a wide range of cytokine receptors [such as interleukin (IL)- 2-, IL- 4-, and IL-10-receptor families besides type I and type II IFN], specific JAK1 inhibitors are under investigation in further inflammatory skin conditions, including atopic dermatitis (AD) (49) and psoriasis (PSO) (50), which demonstrated significant beneficial effects in clinical trials, as well as hidradenitis suppurativa (NCT03569371).

*In vivo* oral treatment with JAK inhibitors ruxolitinib (51) and tofacitinib (52) has been shown to be effective previously on skin lesions in different established lupus prone mouse models. For the treatment of skin disorders, topical drugs which are able to penetrate the skin barrier and provide limited systemic effects are of particular importance. Following the *in vivo* observation of topically applied JAK1 selective INCB039110 leading to a constant improvement of lesional skin with a significantly reduced mouse CLASI score, IFN-regulated gene expression and CD45 + inflammatory cell infiltration in TREX1^–/–^ mice (Figures 4A–D), we emphasize that topically applied JAK1 inhibitors might be an effective alternative to oral compounds for CLE skin lesions. In both interface-dermatitis featuring LP (NCT03697460) and graft-versus-host-disease (NCT03395340), topical formulations of JAK inhibitor ruxolitinib are currently evaluated in phase II clinical trials. In AD, PSO, vitiligo and alopecia areata, topically administered JAK inhibitors have been tested in clinical studies with some promising results concerning safety, tolerability and efficacy, awaiting further investigations (53–56).

## Conclusion

Our data demonstrates that JAK1-specific inhibition significantly decreases the expression of CLE-typical proinflammatory cytokines *in vitro* and *in vivo.* Topical application of a JAK1 selective inhibitor was highly effective in the treatment of CLE-like lesions in lupus prone mice, supporting their potential use in human CLE. Taken together, JAK1 specific inhibitors and, in particular, topical formulations, both preventing partly severe side effects either of JAK2- and JAK3 blockade or oral administration, might be a promising approach for the treatment of LE skin lesions.

## Data Availability Statement

The datasets generated for this study are available on request to the corresponding author.

## Ethics Statement

The studies involving human participants were reviewed and approved by the local Ethics Committee Bonn (BN 09004). The patients/participants provided their written informed consent to participate in this study. The animal study was reviewed and approved by the Animal Welfare Commission of North Rhine-Westphalia, Germany (AZ 2014.A436).

## Author Contributions

TF, TG, and JW performed the research. JW and TF designed the research study. Incyte Corporation kindly contributed JAK1 inhibitor INCB039110. TF and JW analyzed the data and wrote the manuscript. CB and TB contributed essential resources and revised the article critically. All authors contributed to manuscript revision, read, and approved the submitted version.

## Conflict of Interest

This Investigator Sponsored Study was supported by Incyte. PS is employed by the company Incyte Corporation. The remaining authors declare that the research was conducted in the absence of any commercial or financial relationships that could be construed as a potential conflict of interest.
