# Supplementary material for: Selective Janus Kinase 1 Inhibition Is a Promising Therapeutic Approach for Lupus Erythematosus Skin Lesions
Source: Front Immunol. 2020 Mar 3;11:344. doi: 10.3389/fimmu.2020.00344 (PMC7064060; doi:10.3389/fimmu.2020.00344)
Supplement: Supplementary file 1 [file Data_Sheet_1.docx]

Supplementary Material

Selective Janus kinase 1 inhibition is a promising therapeutic approach for lupus erythematosus skin lesions

**Tanja Fetter^1^, Paul Smith^2^, Tugce Guel^1^, Christine Braegelmann^1^,**

**Thomas Bieber^1^, Joerg Wenzel^1^**

^1^Department of Dermatology and Allergy, University Hospital Bonn, Germany

^2^Incyte Corporation, Wilmington, DE, USA

Supplementary Figures

**Supplementary Figure S1. Associated pathways of significantly upregulated genes in CLE lesional skin**. KEGG pathways were classified using Database for Annotation, Visualization and Integrated Discovery (DAVID ver. 6.8) and assigned to innate and adaptive immune system, immunometabolism, disease association and other pathways. Gene counts: number of genes >2-fold upregulated in CLE lesions within the respective KEGG pathway (*p*<0.01).


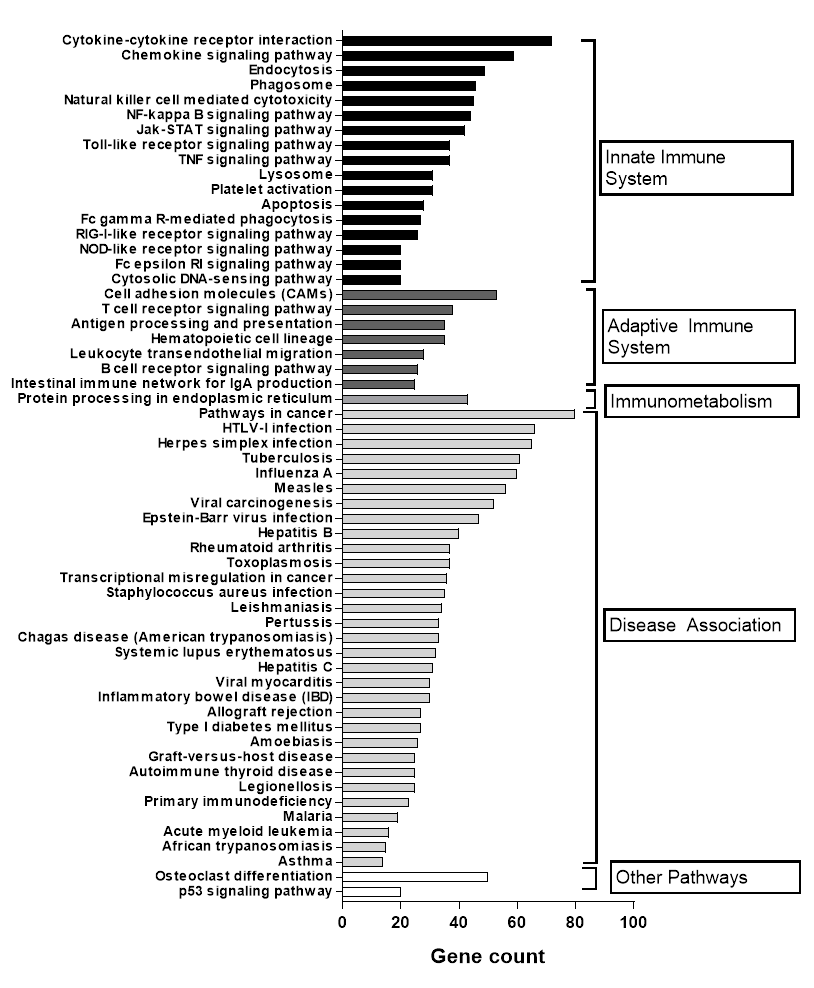


**Supplementary Figure S2. Effect of JAK inhibitors on CXCL10 protein expression in immortalized HaCaT cells, primary epidermal keratinocytes (NHEK) and human epidermis equivalents (epiCS) stimulated with different immunogenic nucleic acids.**


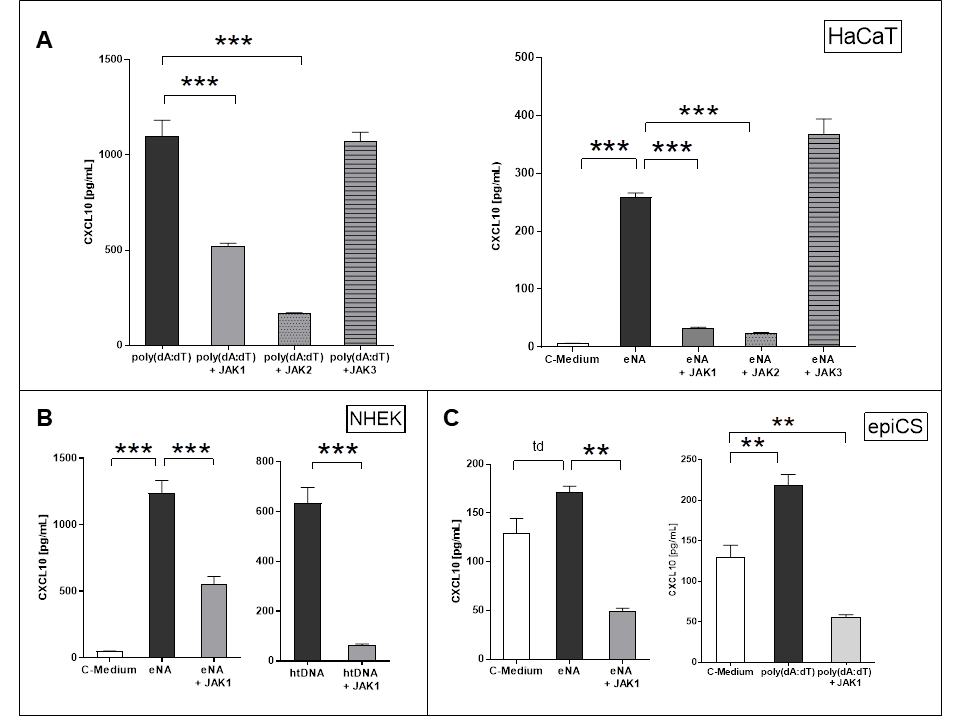


**(A-C**) CXCL10 expression was measured in unstimulated (C-Medium) and stimulated JAK inhibitor treated and untreated keratinocyte cell lines after 24 hours of incubation. Stimulation with immunostimulatory nucleic acids: (a) a synthetic analogue of dsDNA, poly(dA:dT) at a concentration of 1µg/mL, both acquired by InvivoGen, San Diego, USA; (b) endogenous nucleic acids (eNA) isolated from unstimulated cells (using “Genomic DNA from tissue“ kit by Machery-Nagel, Dueren, Germany) and (c) a natural DNA isolated from herring testes (htDNA) at a concentration of 1µg/mL, acquired by Sigma Aldrich Merck KGaA, Darmstadt, Germany. Graphs show effects of JAK1 selective inhibitor INCB039110, JAK2 selective CEP-33779 and JAK3 selective FM-381 on CXCL10 expression in the supernatant of (**A**) HaCaT stimulated with poly(dA:dT) and eNA, (**B**) NHEK stimulated with eNA and htDNA, (**C**) epiCS stimulated with eNA and poly(dA:dT). Measurement by ELISA. All bars show mean + SEM, *** ≙ p<0,001, ** ≙ p<0,01, td ≙ p<0,1 (Mann-Whitney *U* test).

Supplementary Tables

**Supplementary Table 1. Downregulated KEGG pathways in stimulated NHEK after treatment with JAK1 inhibitor INCB039110**. KEGG pathways were classified using Database for Annotation, Visualization and Integrated Discovery (DAVID ver. 6.8). *P*-values were generated with EASE Score. Count: number of genes >2-fold downregulated in NHEK by INCB039110 within the respective KEGG pathway.

| **Pathway** | **KEGG** | ***p*-value** | **Count** |
| --- | --- | --- | --- |
| **Innate Immune System** |  |  |  |
| Cytokine-cytokine receptor interaction | hsa04060 | 4,10E-05 | 9 |
| Chemokine signaling pathway | hsa04062 | 2,20E-02 | 5 |
| Toll-like receptor signaling pathway | hsa04620 | 2,30E-02 | 4 |
| Cytosolic DNA-sensing pathway | hsa04623 | 5,30E-02 | 3 |
| **Immunometabolism** |  |  |  |
| Arachidonic acid metabolism | hsa00590 | 5,40E-03 | 4 |
| alpha-Linolenic acid metabolism | hsa00592 | 9,00E-03 | 3 |
| Linoleic acid metabolism | hsa00591 | 1,20E-02 | 3 |
| Ether lipid metabolism | hsa00565 | 2,80E-02 | 3 |
